# Antagonistic regulatory effects of a single cis-acting expression quantitative trait locus between transcription and translation of the *MRPL43* gene

**DOI:** 10.1186/s12863-022-01057-7

**Published:** 2022-06-04

**Authors:** Jooyeon Han, Chaeyoung Lee

**Affiliations:** Department of Bioinformatics and Life Science, Soongsil University, Seoul, 06978 South Korea

**Keywords:** Expression quantitative trait locus, Functional variant, Mixed model, Mitochondrial ribosomal protein L43, Regulation of gene expression

## Abstract

**Background:**

Heterogeneity of expression quantitative trait locus (eQTL) effects have been shown across gene expression processes. Knowledge on how to produce the heterogeneity is quite limited. This study aims to examine fluctuations in differential gene expression by alleles of sequence variants across expression processes.

**Results:**

Genome-wide eQTL analyses with transcriptome-wide gene expression data revealed 20 cis-acting eQTLs associated simultaneously with mRNA expression, ribosome occupancy, and protein abundance. A 97 kb-long eQTL signal for mitochondrial ribosomal protein L43 (*MRPL43*) covered the gene, showing a heterogeneous effect size on gene products across expression stages. One allele of the eQTL was associated with increased mRNA expression and ribosome occupancy but decreased protein abundance. We examined the heterogeneity and found that the eQTL can be attributed to the independent functions of three nucleotide variants, with a strong linkage. NC_000010.11:g.100987606G > T, upstream of *MRPL43*, may regulate the binding affinity of transcription factors. NC_000010.11:g.100986746C > G, 3 bp from an *MRPL43* splice donor site, may alter the splice site. NC_000010.11:g.100978794A > G, in the isoform with a long 3′-UTR, may strengthen the binding affinity of the microRNA. Individuals with the TGG haplotype at these three variants had higher levels of mRNA expression and ribosome occupancy than individuals with the GCA haplotype but lower protein levels, producing the flipped effect throughout the expression process.

**Conclusions:**

These findings suggest that multiple functional variants in a linkage exert their regulatory functions at different points in the gene expression process, producing a complexity of single eQTLs.

**Supplementary Information:**

The online version contains supplementary material available at 10.1186/s12863-022-01057-7.

## Background

Many quantitative trait loci (QTLs) have been identified from genome-wide association studies (GWAS) for complex phenotypes over the last decade, but the understanding of their underlying functions is mostly vague [1]. The genetics of gene expression is critical in understanding gene regulation with the QTLs and dissecting the genetic basis of complex phenotypes. Genome-wide expression quantitative trait loci (eQTLs), especially cis-eQTLs, account for a substantial proportion of variation in gene expression [2]. Furthermore, this genome-wide eQTL analysis incorporating transcriptome-wide expression data may provide the regulatory genetic architecture of every gene in a human cell [3].

A variety of genome-wide identifications of eQTLs have been provided by layers of gene regulation. Comparison of the data might help in understanding the specific function during each expression stage. For example, when a genome-wide association study was conducted to identify mRNA expression QTL (neQTL: narrow-sense eQTL), ribosome occupancy eQTL (rQTL), and protein abundance eQTL (pQTL), a nucleotide near the 3′-UTR, NC_000022.11:g.36209931A > T, was found to be significant not as an neQTL or rQTL, but as a pQTL for the apolipoprotein L2 (*APOL2*) gene [4]. An acetylation site in proximity to the protein-specific QTL implied a regulatory function of lysine acetylation in the degradation of the protein. Similar to this protein-specific QTL, many eQTLs (71%; 46% neQTL, 16% rQTL, and 9% pQTL) were identified only once from the three kinds of data [4]. Among the stage-specific eQTLs, it is difficult to filter out spurious eQTLs produced by experimental errors or confounding. Replications of the stage-specific eQTLs are needed to avoid false positives and to confirm expressional regulations.

The effect sizes of eQTLs showed fluctuations across the regulation stages. In particular, the effect size of the pQTL decreased compared with those of the neQTL and rQTL.

This post-transcriptional buffering effect appeared in many genes [4]. This was explained as a negative feedback regulation of the gene itself to reduce differential transcription produced by nucleotide variants [5]. More recently, it has also been treated as an adaptational regulation of translation rates to maintain balance in protein levels [6, 7]. The buffering effect helps maintain homeostatic steady-state protein levels [8–10]. Producing this difference and reducing it by negative feedback regulation might be considered a fundamentally inefficient mechanism. Understanding the genetics underlying control of protein abundance is important because it is the direct determinant of cellular function as the final product of gene expression [11]. It is crucial to understand how protein abundance is determined by various expression controls to understand the underlying mechanisms of specified eQTLs. Nevertheless, few attempts to identify differences in effect size have been made aside from studies on the buffer effects. The heterogeneous effect size of eQTLs might be strongly attributed to spatial and temporal regulation in its specific function. However, multiple functions of eQTLs are also suspected to produce this heterogeneity.

The aims of this study are to examine fluctuations in differential gene expression by alleles of nucleotide variants simultaneously associated with mRNA expression, ribosome occupancy, and protein abundance, and to uncover their multiple regulatory functions across expression stages. We employed a mixed model to adjust genetic backgrounds in the genome-wide eQTL analysis. We revealed the complexity of the gene regulation of mitochondrial ribosomal protein L43 (*MRPL43*) caused by multiple functional variants in strong linkage.

## Results

We identified 84,094, 31,933, and 12,690 associations of nucleotide variants with mRNA expression, ribosome occupancy, and protein abundance, respectively (*P* < 1 × 10^− 5^). Of these, 117 were shared by mRNA expression, ribosome occupancy, and protein abundance. These turned out to be 20 eQTL signals, each located in an LD block constructed by the algorithm developed by Gabriel et al. [12]. All were located in and around the corresponding gene; 19 eQTLs were found for the major histocompatibility complex, class II, DQ alpha 1 (*HLA-DQA1*) gene, and one was for the *MRPL43* gene. The eQTLs for *HLA-DQA1* had a range of 32,603,487–32,658,801 bp (hg19) in chromosome 6, including 503 nucleotide variants (Online Resource Fig. S1). Although only one eQTL signal was identified for *MRPL43*, this had a wider range, from 102,670,196 to 102,767,155 bp in chromosome 10 (hg19), including 41 nucleotide variants. These cis-acting eQTLs are presented with their representative nucleotide variants and significances for associations with mRNA expression, ribosome occupancy, and protein abundance in Table 1.

**Table 1:** Nucleotide variants associated with mRNA expression, ribosome occupancy, and protein abundance of HLA-DQA1 and MRPL43^a^

SNP	Position^b^	MAF	mRNA expression		Ribosome occupancy		Protein abundance	
			BETA	P	BETA	P	BETA	P
HLA-DQA1								
g.32637603 T > A	6:32,605,380	0.48	0.842	2.78 × 10^−8^	0.614	7.15 × 10^− 6^	0.886	1.50 × 10^−7^
g.32639416 T > C	6:32,607,193	0.24	−0.678	7.83 × 10^−7^	− 0.647	4.48 × 10^− 7^	− 0.741	6.15 × 10^−6^
g.32639504G > A	6:32,607,281	0.36	−0.573	2.87 × 10^−6^	−0.501	5.75 × 10^−6^	−0.637	2.72 × 10^−6^
g.32640436G > A	6:32,608,213	0.44	−0.687	2.17 × 10^−7^	−0.537	6.91 × 10^−6^	−0.692	2.58 × 10^−6^
g.32641103G > A	6:32,608,880	0.27	−0.790	7.69 × 10^−8^	−0.716	1.90 × 10^−7^	−0.881	7.70 × 10^−8^
g.32641737C > A	6:32,609,514	0.48	0.840	3.05 × 10^−8^	0.607	8.83 × 10^−6^	0.873	2.24 × 10^−7^
g.32644006A > G	6:32,611,783	0.40	−0.628	7.63 × 10^−7^	− 0.533	3.34 × 10^−6^	− 0.709	3.91 × 10^− 7^
g.32652582C > A	6:32,620,359	0.37	−0.597	1.42 × 10^−6^	−0.495	9.22 × 10^−6^	−0.725	1.38 × 10^−7^
g.32658175C > A	6:32,625,952	0.47	−0.725	1.48 × 10^−7^	−0.567	6.87 × 10^−6^	−0.770	1.07 × 10^−6^
g.32658472 T > A	6:32,626,249	0.45	−0.757	2.86 × 10^−7^	−0.676	6.63 × 10^−7^	−0.874	4.65 × 10^−8^
g.32658813C > A	6:32,626,590	0.48	0.856	3.08 × 10^−8^	0.632	5.87 × 10^−6^	0.916	1.19 × 10^−7^
g.32661067 T > A	6:32,628,844	0.43	−0.638	9.91 × 10^−7^	− 0.553	3.24 × 10^−6^	− 0.649	8.98 × 10^− 6^
g.32661176C > A	6:32,628,953	0.39	−0.641	2.82 × 10^−7^	−0.505	8.44 × 10^−6^	−0.656	3.23 × 10^−6^
g.32662025A > C	6:32,629,802	0.52	0.841	2.62 × 10^−8^	0.634	3.22 × 10^−6^	0.904	1.66 × 10^−7^
g.32669003G > A	6:32,636,780	0.44	−0.746	2.88 × 10^−7^	− 0.642	1.60 × 10^−6^	− 0.848	6.94 × 10^−8^
g.32669230G > C	6:32,637,007	0.42	−0.708	5.50 × 10^−7^	−0.659	4.22 × 10^−7^	−0.802	1.38 × 10^−7^
g.32670046A > G	6:32,637,823	0.40	−0.674	9.23 × 10^−7^	−0.650	2.83 × 10^−7^	−0.799	1.11 × 10^−7^
g.32670110 T > C	6:32,637,887	0.42	−0.701	5.72 × 10^−7^	−0.612	2.16 × 10^−6^	−0.788	2.57 × 10^−7^
g.32670309G > A	6:32,638,086	0.41	−0.729	1.59 × 10^−7^	−0.639	6.07 × 10^−7^	−0.750	7.31 × 10^−7^
MRPL43								
g.100983006C > A^c^	10:102,742,763	0.47	0.534	9.16 × 10^−6^	0.748	7.47 × 10^−8^	−0.577	6.09 × 10^−6^
g.100986746C > G^c^	10:102,746,503	0.47	0.534	9.16 × 10^−6^	0.748	7.47 × 10^−8^	−0.577	6.09 × 10^−6^
g.100980514 T > C^c^	10:102,740,271	0.47	0.534	9.16 × 10^−6^	0.748	7.47 × 10^−8^	−0.577	6.09 × 10^−6^

^a^Only representative nucleotide variants are presented (P < 1 × 10^−5^)

^b^Chromosome number: chromosomal position in the hg19 version

^c^The three nucleotide variants in complete linkage had the lowest *P* value in one signal

**Fig. 1:**
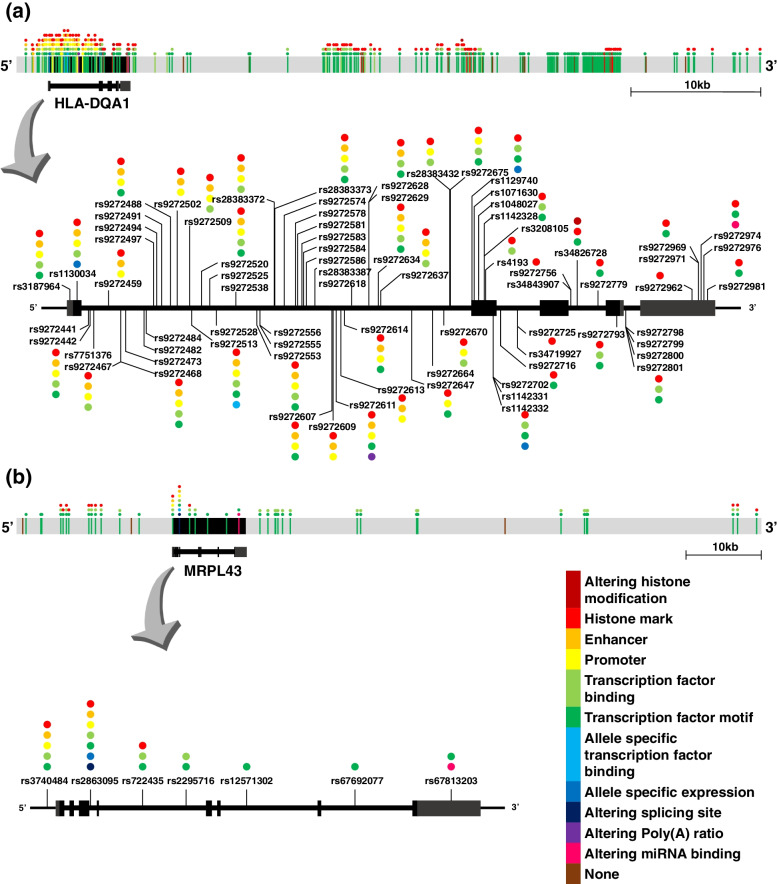
Functional nucleotide variants within the eQTL signals for *HLA-DQA1* (**a**) and *MRPL43* (**b**). Dots with a variety of colors indicate functions of the nucleotide variants as presented in the index bar. Line color of the nucleotide variant indicates the corresponding function shown at the last expression stage. Black boxes indicate exons. Chromosomal position is relative to the human reference sequence hg19

The *HLA-DQA1* expression increased with a certain allele of its eQTL and decreased with the other allele regardless of mRNA expression, ribosome occupancy, and protein abundance. A variety of functions of the nucleotide variants were found across the eQTL region, and eQTLs with likely functions are presented in Fig. 1a. Two nucleotide variants likely affecting histone modification were uncovered by exploring ChIP-seq data obtained from the Roadmap Epigenomics study: NC_000006.12:g.32642332A > C using H3K4me1 and H3K4me3; and NC_000006.12:g.32668657A > G using H3K4me1. HaploReg showed several transcription factor binding sites around the transcription start site, which were identified by ChIP-Seq against transcription factors. Potential allelic imbalance in transcription factor binding between homologous chromosomes of heterozygous individuals of the 1000 Genomes Project was found for two nucleotide variants (T:G = 30:0 for NC_000006.12:g.32638603 T > G and C:A = 27:1 for NC_000006.12:g.32638840C > A) in intron 1 of *HLA-DQA1*. Many significant consensus sequences altered by the nucleotide substitution were found by the ENCODE project. Exon-specific association analysis using the paired-end 75 bp mRNA-seq data obtained by Lappalainen et al. [13] also revealed the allelic imbalance in *HLA-DQA1* expression between homologous chromosomes of heterozygous individuals (*P* < 1 × 10^− 5^). A significant poly(A) ratio was found between the alleles of NC_000006.12:g.32640003C > A in intron 1 of *HLA-DQA1* to likely alter the poly(A) site (*P* = 3.27 × 10^− 310^). The miRDB database predicted that some 3′-UTR nucleotide variants (NC_000006.12:g.32643538C > T and NC_000006.12:g.32643564G > A) may be associated with miRNA binding affinity.

**Fig. 2:**
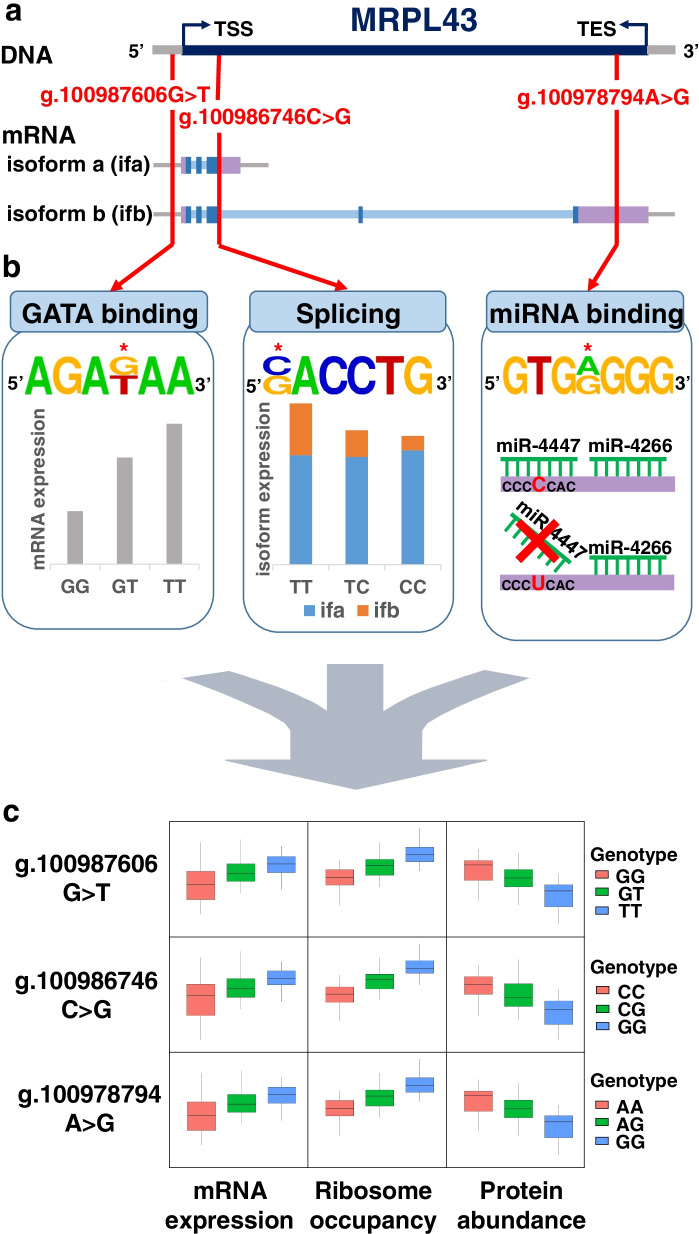
Example of various functions of multiple nucleotide variants in the strong linkage of the eQTL signal for *MRPL43*. Positions of nucleotide variants in DNA and RNA (**a**), functions of the nucleotide variants marked with an asterisk (**b**), expression effects resulting from the functions (**c**). Human reference sequence hg19 was used for consensus sequences. An asterisk indicates a nucleotide variant with major (top) and minor (bottom) alleles. Note that the GATA in (**b**) is presented as a candidate transcription factor that can cause differential binding affinity and might cause differential transcription by allele substitution

In the large eQTL for *MRPL43*, the A allele of the NC_000010.11:g.100983006C > A or linked alleles were associated with increased mRNA expression and ribosome occupancy and with decreased protein abundance. Further analysis also showed various potential functions of the nucleotide variants within the eQTL, as shown in Fig. 1b. The analysis revealed that the difference in expression of *MRPL43* across expression stages could be attributed to independent functions of nucleotide variants within its eQTL (Fig. 2). One nucleotide variant (NC_000010.11:g.100987606G > T; rs3740484) 87 bp upstream of *MRPL43* was located in a transcription factor binding site uncovered by the ChIP-seq data with RNA polymerase and relevant components resulting from the ENCODE Project. The promoter function was supported by a variety of epigenomic data with chromatin states obtained from the Roadmap Epigenomics Consortium (Core 15-state model, 25-state model with 12 imputed marks, H3K4me1, H3K4me3, H3K27ac, K3K9ac, and DNase). This variant can alter the recognition site for GATA, and its T allele increased binding affinity to GATA 2.95–8.67 times (HaploReg 4.1). Another variant (NC_000010.11:g.100986746C > G; rs2863095), 3 bp downstream from the splice donor site of exon 3, may alter the splice site and thus produce an isoform of *MRPL43*. Exon-specific analysis for mRNA expression revealed that the G allele of NC_000010.11:g.100986746C > G increased long transcripts with exons 4, 5, 6, and 7 (*P* < 1 × 10^− 5^).

Isoform-specific analysis for mRNA expression showed more transcripts with a long 3′-UTR in individuals with the G allele (P < 1 × 10^− 5^), and allelic imbalance in heterozygous individuals was also observed for the nucleotide variant. Further analysis using SpliceAid2 identified a splicing factor, zinc finger ran-binding domain-containing protein 2 (ZRANB2), that likely binds to the G allele of NC_000010.11:g.100986746C > G, but not to its C allele. A variant, NC_000010.11:g.100978794A > G, within the long 3′-UTR was specific for this isoform and was located in the 7-mer seed sequence for microRNA binding. The miRDB showed that miR-4447 microRNA bound with its G allele, but not with its A allele.

Deep learning analyses supported that all the promoter (NC_000010.11:g.100987606G > T), intronic (NC_000010.11:g.100986746C > G), and 3′-UTR (NC_000010.11:g.100978794A > G) nucleotide sequence variants could contribute to the expression of *MRPL43* with independent functions across the expression stages. ExPecto predicted that transcription of *MRPL43* was affected by the promoter variant, but not by the intronic or 3′-UTR variant. SpliceAI yielded a splice donor 3 bp upstream of the intronic variant. The probability increased by 0.46 when its allele was substituted from C to G. miTAR predicted the miRNA of has-miR-4447 and its target, 3′-UTR of *MRPL43*. The calling probability decreased with the A allele (0.87) of the 3′-UTR variant compared with that with the G allele (0.98).

## Discussion

The current genome-wide eQTL analysis with transcriptome-wide data revealed cis-acting eQTLs for *HLA-DQA1* and *MRPL43* by employing a mixed model, showing associations with mRNA expression, ribosome occupancy, and protein abundance. All eQTLs included many potentially functional nucleotide variants in strong linkage over a wide range.

We found only one eQTL for *MRPL43*; this had flipped effects across expression stages, implying its involvement in multiple functions. This eQTL covering the gene was 96,960 bp long, and a variety of functional nucleotide variants were identified within it. For example, Fig. 2 shows three nucleotide variants in linkage with different functions, especially at different expression regulatory stages. NC_000010.11:g.100987606G > T, a nucleotide variant in the promoter of *MRPL43*, might alter the binding affinity to transcription factors such as GATA, a transcription factor binding site. NC_000010.11:g.100986746C > G, a nucleotide variant next to the splice donor site of exon 3, altered a splice site, which was likely to result in the production of an isoform of *MRPL43*. The NC_000010.11:g.100978794A > G, a nucleotide variant of a 7-mer microRNA binding site for miR-4447 in its 3′-UTR, controlled translation. We found that 94.7% of the Yoruba population was composed of two major haplotypes (GCA and TGG) of these three variants (NC_000010.11:g.100987606G > T, NC_000010.11:g.100986746C > G, and NC_000010.11:g.100978794A > G). Thus, an end product can be determined by summing up all the effects of these variants in different stages of gene expression. Individuals with the T allele of NC_000010.11:g.100987606G > T have higher mRNA levels because of the enhanced transcription factor binding affinity of the T allele. This is consistent with results from a previous study where the substitution of the T allele to a G allele in the GATA consensus sequence undermined GATA binding and gene expression [13]. The individuals with the G allele of NC_000010.11:g.100986746C > G in strong linkage with the T allele of NC_000010.11:g.100987606G > T show nearby splicing more frequently through enhanced recognition of the G allele over the C allele by the splicing factor ZRANB2. As a result, these individuals have more specific isoforms with long 3′-UTRs. In general, mRNAs with a long 3′-UTR appear to be less stable than those with a short 3′-UTR. In particular, the G allele of NC_000010.11:g.100978794A > G within the long 3′-UTR in strong linkage with the G allele of NC_000010.11:g.100986746C > G is a critical nucleotide of the miRNA binding site. The nucleotide can enhance the binding affinity and specificity as the fifth nucleotide of the miRNA binding sequence as shown in previous studies where mRNA sequence pairing with the nucleotides 2–8 of the miRNA played a central role in binding to the miRNA bound by Argonaute [14]. This miRNA binding site has the important function of interfering with translation considering another miRNA binding site in proximity. Such multiple miRNA binding sites are considered to greatly destabilize mRNA [15]. This interference might be crucial to the isoform in producing protein, even contributing to the flipping effect. This flipping effect shows that it is the result of active control not passive control, unlike the buffer effect. The substantial control by the interference concurs with previous studies [16, 17], in which elongation speed of translation was considerably controlled for ribosomal proteins.

*MRPL43*, a nuclear gene, encodes a component of the large subunit of the mitochondrial ribosomal protein (MRP) and plays a core role in synthesizing proteins in the mitochondrion. The MRP is critical in mitochondrial dysfunction and some pathological conditions [18]. In particular, impaired translation in mitochondria may result in many phenotypic abnormalities, including hypertrophic cardiomyopathy, psychomotor retardation, growth retardation, and neurological deterioration [19–21]. A possibility under consideration is that the genetic variants responsible for regulating the expression of *MRPL43* might influence these phenotypes or their intermediate products. For example, individuals with the second most frequent haplotype (TGG of the functional variants) of eQTL for *MRPL43* exhibited reduced protein levels at the final stage as shown in the current study. This is a potential factor associated with susceptibility to diseases. Further studies are required to examine the contribution and the interaction with other factors.

The promoter variant was found in a transcription factor binding site via the ChIP-seq experiments with RNA polymerase and relevant components and by various regulatory chromatin states with histone marks and DNase. Thus, the binding affinity of the variant to some transcription factors differs by its allele substitution. For example, a stronger binding affinity (3.0–8.7 times) of its T allele to GATA was estimated based on a position frequency matrix. Experimental investigation is needed to confirm the influence of the GATA binding to the promoter variant NC_000010.11:g.100987606G > T on transcribing the *MRPL43*. Likewise, specifically designed experiments would support the other causative variants, NC_000010.11:g.100986746C > G and NC_000010.11:g.100978794A > G, in splicing and microRNA binding, respectively.

Furthermore, this study found several eQTLs in and around the *HLA-DQA1* gene. Many nucleotide variants in this large region are in strong linkage. Furthermore, they are complexly linked to nucleotide variants outside, especially within the major histocompatibility complex. This necessitates a careful interpretation of functional variants, especially in assessing the effect size of functional variants. Thus, studies with sophisticated design are required to identify functional variants with heterogeneous effects over different expression stages.

Because this study only dealt with the eQTLs simultaneously associated with mRNA expression, ribosome occupancy, and protein abundance, we did not examine regulatory functions of eQTLs associated with only one or two of them which might be caused by multiple functional variants in linkage. eQTLs identified at an early stage might act antagonistically with the nucleotide alleles that compose a specific haplotype, and thus the effects produced by the eQTLs disappear at a later stage by the antagonistic function. Such a disappearance is more likely observed as a buffering effect. In terms of genetics and evolution, the antagonistic function should be distinguished from the buffering effect. The antagonism is an active mechanism by genetic variants, and the buffering is a negative feedback mechanism for homeostatic maintenance of protein levels.

Genotype imputation is considered an important process that can infer missing genotypes of nucleotide variants linked with known markers based on their linkage disequilibrium in a reasonable reference population. This enables us to identify more GWAS signals and integrate multiple studies for meta-analysis [22]. However, false genotypes produced by imputation may lead to bias in eQTL effect size. We conducted eQTL analysis without any imputation of genotypes in the current study to avoid such biases because this study considered eQTL effect size rather than eQTL discovery.

The current study employed a mixed model with polygenic covariance among individuals to identify eQTLs. The mixed model approach helps avoid spurious eQTLs, which might be produced by population stratification [23]. The best linear unbiased estimates of eQTL effects using the mixed model were used to determine their identification [24]. Accuracy is crucial in the current eQTL analysis. This study focused not only on the identification of eQTLs but also the comparison of eQTLs in terms of expression products and stages to determine their functions.

## Conclusions

The current genome-wide analysis revealed eQTL signals for *MRPL43* and *HLA-DQA1*, showing associations with mRNA expression, ribosome occupancy, and protein abundance. Heterogeneity was shown in their effect sizes across the stages of gene expression. A variety of functions across expression stages were identified within each signal. This study suggests that an end product of gene expression could be summed up by the individual functional effects of nucleotide variants. The eQTL for *MRPL43* is a good example with multiple functions by different nucleotide variants in strong linkage, even showing a flipped effect. Many eQTLs associated with one or two of the parameters for mRNA expression, ribosome occupancy, and protein abundance in this study may have been caused by multiple functional variants in linkage. In particular, eQTLs identified at an early stage may have an antagonistic function with the nucleotide alleles that compose a specific haplotype. Considering that many eQTLs generally have many nucleotide variants in linkage, research efforts on the decomposition and quantification of individual functions are required to understand the underlying mechanism of differential gene expression and their roles in complex phenotypes.

## Methods

### Subjects and expression data

eQTL analysis was first conducted using expression data of mRNAs, ribosome occupancy, and proteins from lymphoblastoid cell lines (LCLs) of 63 Yoruba individuals in Ibadan, Nigeria who had participated in the HapMap project. We used high resolution mRNA expression data produced by Pickrell et al. [25, 26]. They sequenced cDNA libraries for the RNA with polyadenylation from each individual in at least two lanes of the Illumina Genome Analyzer 2 platform and mapped reads to the human genome using MAQ v0.6.8. They had a median coverage of 8.6 million mapped reads per sample. We used ribosome occupancy data as an index of intermediate regulations between transcription and post-translation. The data were quantified by Battle et al. [4] using the ARTseq Ribosome Profiling kit for mammalian cells (RPHMR12126) and had a median of 12.1 million mapped reads per individual. Both mRNA expression and ribosome profiling data were calculated as the sum of reads per kilobase per million mapped reads for all transcripts of each gene in each individual. We used protein abundance data calculated as relative values to a SILAC internal standard sample (i.e., ${\log}_2\;\frac{\mathrm{sample}}{\mathrm{standard}}$) produced by quantitative protein mass spectrometry [4].

This analysis excluded all genes with three or more missing samples. mRNA expression, ribosome occupancy, and protein abundance were independently standardized and quantile-normalized to reduce technical variation among the data sets [27]. Principal component analysis was then conducted to reduce the impact of hidden confounders from all the data sets of mRNA expression, ribosome occupancy, and protein abundance. Six, nine, and seven principal components were regressed out to maximize the number of eQTLs.

The corresponding genotypic data were obtained from the study of the 1000 Genomes Project Consortium [28], in which low-coverage whole-genome sequencing, deep exome sequencing, and dense microarray genotyping were used. Nucleotide variants with minor allele frequency < 0.1 or with Hardy-Weinberg disequilibrium (*P* < 1 × 10^− 6^) were removed. Only individuals with both genotypes and the specific molecular level were included in the corresponding analysis. In the current study, 63 individuals were analyzed for mRNA expression, 62 for ribosome profiling, and 51 for protein abundance.

### Statistical methods

To discover eQTLs, we employed a mixed linear model that included random polygenic effects to explain the variability of individual genetic backgrounds. The polygenic variability can be estimated by the covariance structure of pairwise genomic similarity among individuals, based on the genotype information of genome-wide nucleotide variants. This avoids population stratification and explains the remaining genetic effects aside from the candidate locus, and as a result, false-positive associations can be reduced [29].

The analytical model employed in the current study was as follows:

$$\boldsymbol{y}=\boldsymbol{x}\upbeta +\boldsymbol{g}+\boldsymbol{\varepsilon}$$

where ***y*** is the vector (n × 1) of the gene expression levels, n is the number of the gene expression levels, β is the scalar of the fixed minor allele effect of the candidate nucleotide variant, ***x*** is the design vector (n × 1) for the fixed effect, ***g*** is the vector (n × 1) of random polygenic effects, and ***ε*** is the vector (n × 1) of random residuals. Elements of the vector x are classified as the number of minor alleles (0, 1, or 2) under the assumption of an additive genetic model. The random variables ***g*** and ***ε*** in the analytical model have the following normal distributions:

$$\bf {\displaystyle \begin{array}{c}\boldsymbol{g}\sim N\left(\mathbf{0},\boldsymbol{G}{\sigma}_g^2\right)\\ {}\boldsymbol{\varepsilon} \sim N\left(\mathbf{0},\boldsymbol{I}{\sigma}_{\varepsilon}^2\right)\end{array}}$$

where ${\sigma}_g^2$ is the polygenic variance component, ${\sigma}_{\varepsilon}^2$ is the residual variance component, ***I*** is the identity matrix (n × n), and ***G*** is the n × ngenomic similarity matrix (n × n) with elements of pairwise genomic similarity coefficients based on genotypes of nucleotide variants. The genomic similarity coefficient (*g*_*jk*_) between individuals *j* and *k* can be calculated as follows [29]:

$${g}_{jk}=\frac{1}{n_v}\sum_{i=1}^{n_v}\frac{\left({\tau}_{ij}-2{f}_i\right)\left({\tau}_{ik}-2{f}_i\right)}{2{f}_i\left(1-{f}_i\right)}$$

where *n*_*v*_ is the number of nucleotide variants that contribute to the genomic similarity, *τ*_*ij*_ and *τ*_*ik*_ are the numbers (0, 1, or 2) of minor alleles for the nucleotide variant *i* of the individuals *j* and *k*, and *f*_*i*_ is the frequency of the minor allele. Polygenic and residual variance components were estimated using restricted maximum likelihood (REML). The REML estimates were first obtained by the expectation-maximization (EM) algorithm, then the final REML estimates were obtained by the average information algorithm with the EM-REML estimates as initial values. The nucleotide variant effect was estimated and tested given the variance component estimates. Multiple testing adjusted by permutation was employed to determine significant associations, and a conservative significance threshold value of 1 × 10^− 5^ was applied to the shared eQTL identification. The statistical analyses were conducted using the GCTA program [30]. Nucleotide variants with significant association were determined as eQTLs if they were independent signals. Linkage disequilibrium (LD) blocks at association signals were constructed using Haploview [31].

### Functional analysis

The eQTLs identified from genome-wide association analyses were further examined to identify their functional roles. The functional roles were searched sequentially across expression stages. The eQTLs were examined to find the corresponding methylation sites using genome-wide analyses to identify the association of CpG-sites with their methylation levels observed by the Illumina HumanMethylation27 and Illumina Human Methylation 450 K [32, 33]. The eQTLs were investigated to discover their histone marks using genome-wide chromatin profiles based on H3K4me3, H3K4me1, and H3K27ac produced by LCL-specific Hi-C and ChIA-PET [34]. Epigenomic data including ChromHMM, histone modification ChIP-seq, and DNase hypersensitivity resulting from the Roadmap Epigenomics study [35] were also utilized to find relevant functions of eQTLs.

Regulatory protein-binding sites were examined using the ChIP-seq data with RNA polymerase components in various cell types from the ENCODE Project [36], and the data processed using the narrowPeak algorithm were made publicly available in HaploReg v4 [37]. To examine the effects of the nucleotide variants on protein binding, the position weight matrices were estimated by combining data collected from TRANSFAC [38], JASPAR [39], and other protein-binding microarray experiments [40–42]. To investigate allele-specific binding, we used allelic imbalance measurements between homologous chromosomes of heterozygous individuals using ChIP-seq [43]. The regulatory role of enhancers was also examined using genome-wide integration of enhancers and target genes using the GeneHancer database [44].

Subsequent analysis was conducted for association with expression data of isoforms, exons, or alleles. We used data for isoform-, exon-, and allele-specific transcripts mapped with Genome Multitool mapper using paired-end 75 bp mRNA-seq data obtained using the Illumina HiSeq 2000 platform [13]. The data were made available after quality assurance by sample correlations and removal of technical variation by normalization.

To identify other post-transcriptional functions, the poly(A)-specific transcripts were compared as the poly(A) ratios of at least two poly(A) sites produced from a gene [45]. RNA decay rates obtained from a study with a time-course design were also compared by the alleles of eQTLs [46]. Splicing sites were predicted with intragenic nucleotide variants using RNA sequences bound by splicing proteins in the database of SpliceAid2 [47].

Translational regulatory functions were examined for the eQTLs with the role of regulating the expression of miRNA. We used miRNA expression data produced using the Illumina HiSeq 2000 platform with single-end 36 bp small-RNA-seq [13]. Associations of eQTLs with the abundance of aminoacyl-tRNA synthetase were examined to see whether tRNA shortage functioned as an obstacle to translation, using aminoacyl-tRNA synthetase quantified by high-resolution mass spectrometry [4]. MicroRNA target sequences in the 3′-UTR were predicted using high-throughput profile data made available at miRDB that resulted from the crosslinking and immunoprecipitation followed by RNA ligation studies [48].

The eQTLs identified with potential functions were further investigated by predicting their functions using an artificial intelligence approach (deep learning-based methods). We employed ExPecto to predict the transcriptional effects of nucleotide sequence variants. ExPecto enabled us to predict cell type-specific effects (218 tissues and cell types) of each nucleotide variant based on 2002 different profile data of histone marks, transcription factor binding sites, and DNA accessibility [49]. Splice-altering consequences were predicted employing the SpliceAI [50], a deep neural network algorithm. miRNAs and their targets were predicted using miTAR with DeepMirTar and miRAW datasets. This was devised based on both convolutional and recurrent neural networks to increase prediction accuracy [51].

## Supplementary Information

**Additional file 1: Supplementary Figure 1.** Linkage disequilibrium blocks for nucleotide variants in eQTLsignals for HLA-DQA1 (**A**) and MRPL43 (**B**).

## Data Availability

The data used in this study are publicly available in GEO DataSets (Accession No. GSE61742).

## Abbreviations

eQTL: Expression quantitative trait locus
MRPL43: Mitochondrial ribosomal protein L43
GWAS: Genome-wide association studies
pQTL: Protein abundance eQTL
