# Supplementary material for: Antagonistic regulatory effects of a single cis-acting expression quantitative trait locus between transcription and translation of the MRPL43 gene
Source: BMC Genom Data. 2022 Jun 4;23:42. doi: 10.1186/s12863-022-01057-7 (PMC9167510; doi:10.1186/s12863-022-01057-7)
Supplement: Supplementary file 1 — Additional file 1: Supplementary Figure 1. Linkage disequilibrium blocks for nucleotide variants in eQTLsignals for HLA-DQA1 (A) and MRPL43 (B). [file 12863_2022_1057_MOESM1_ESM.pdf]

(A)

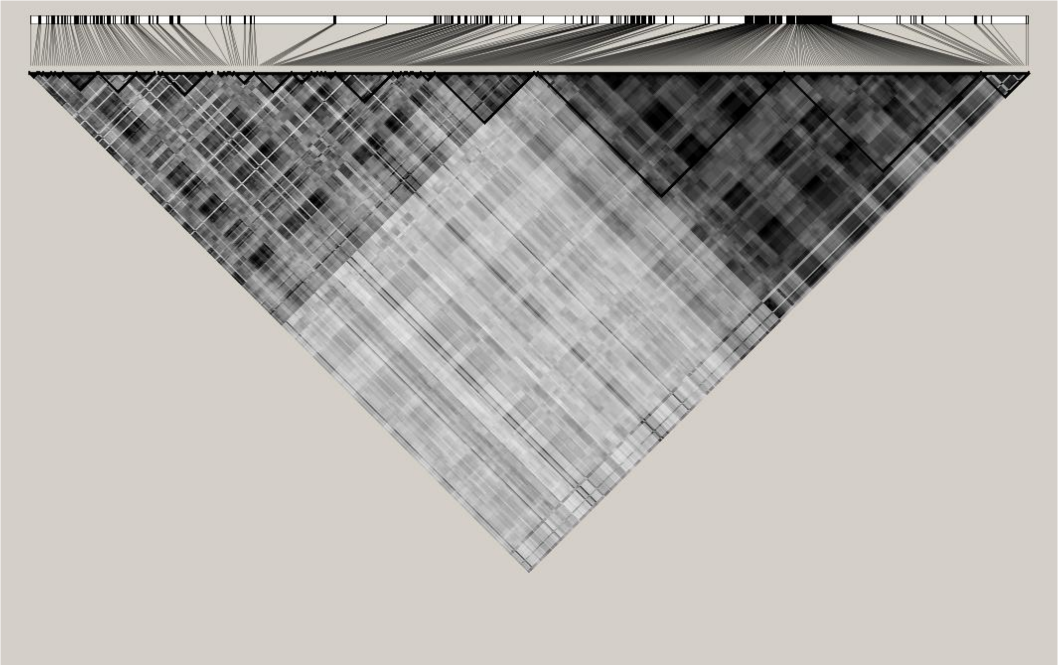

(B)

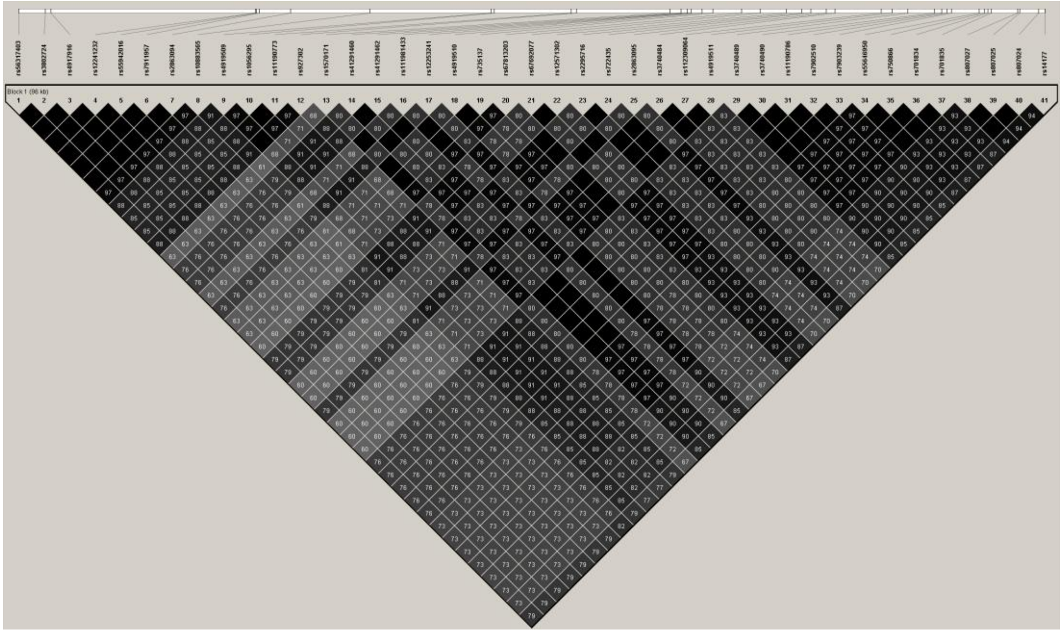

Supplementary Figure 1. Linkage disequilibrium blocks for nucleotide variants in eQTL signals for HLA-DQA1 (**A**) and MRPL43 (**B**).
